# Ulnar nerve thickness at the elbow on longitudinal ultrasound view in control subjects

**DOI:** 10.1186/s42466-023-00230-2

**Published:** 2023-01-26

**Authors:** José Manuel Pardal-Fernández, Inmaculada Diaz-Maroto, Tomás Segura, Carlos de Cabo

**Affiliations:** 1 Department of Clinical Neurophysiology, University General Hospital, C/Hnos. Falcó, 37, 02008 Albacete, Spain; 2 Unit of Neuromuscular Disorders, Department of Neurology, University General Hospital, Albacete, Spain; 3 Department of Neurology, University General Hospital, Albacete, Spain; 4 Neuropsychopharmacology Unit, University General Hospital of Albacete, Albacete, Spain

**Keywords:** Ulnar neuropathy at the elbow, Electrophysiology, Nerve conduction studies, Nerve entrapment, Ultrasonography, Longitudinal ultrasound, Nerve, Ultrasound

## Abstract

**Introduction:**

Ulnar mononeuropathy at the elbow is the second most frequent neuropathy in humans. Diagnosis is based on clinical and electrophysiological criteria and, more recently, also on ultrasound. Cross-sectional ultrasound is currently the most valued, although longitudinal ultrasound allows assessment of the entire affected trajectory of the nerve in a single view, but always in a straight line with no changes in direction, as in the extended elbow. The main aim of this work is to propose normative values ​​for longitudinal ultrasound of the ulnar nerve at the elbow.

**Methods:**

The neurological exploration of upper extremity, and electrophysiological and ultrasound parameters at the elbow of ulnar nerve were evaluated in 76 limbs from 38 asymptomatic subjects.

**Results:**

The diameters of the nerve as well as the distal and proximal areas were larger at the proximal region of the ulnar groove, and even more so in older individuals. In most of these elderly subjects, we found a small, non-significant slowdown in motor conduction velocity at the elbow with respect to the forearm (less than 5 m/s).

**Conclusions:**

We observed a good correlation between the longitudinal and cross-sectional ultrasounds of the ulnar nerve at the elbow. Longitudinal ultrasound proved to be sensitive, reliable, simple and rapid, but its greatest contribution was allowing the visualization of the entire nerve trajectory in an integrated way, providing an image with good definition of the outline, proportions and intraneural characteristics of the nerve.

## Introduction

Ulnar neuropathy at the elbow (UNE) is the second most common neuropathy in humans. Diagnosis is made by clinical signs and symptoms, and is confirmed and characterized by electrodiagnostic tests (EDX) [1].

Ultrasonography (US) is an emerging technique for the diagnosis of neuropathies that has proven to be quite useful [2–9]. The US has been incorporated as a complementary diagnostic procedure for UNE [10–19], especially the cases with confusing or inconclusive clinical findings and EDX [20–22]. It has been established that demographic characteristics such as height, body mass index, age, sex and dominance do not seem to have a significant influence on diagnostic parameters of UNE, and we assume that they do not skew the determination of cut-off points for cross-sectional area (CSA) neural trajectories studied [23–27] except for children [28].

Relevant issues are the small differences in size between the normal and pathological nerves, as well as the discrepancy between the theoretical resolution values and the values ​​obtained with a linear probe. All this may be corrected by establishing uniform demographic criteria.

The optimal place to determine the maximum US thickening in UNE is the segment of the nerve running through the ulnar groove (UG) (between the medial epicondyle (ME) of the humerus and olecranon), which is a path without any obstacle to neural growth. The maximum narrowing may be assessed at the distal segment running through the ulnar tunnel, defined by the passage between the 2 bellies of the *flexor carpi ulnaris* and Osborne’s ligament (roof). Both signs can be visualized with a single longitudinal US evaluation, which may represent an illustrative, integrated, efficient and fast way to diagnose UNE. The main US diagnostic parameter is the CSA at the UG or retroepycondilar groove [2, 3, 12, 13, 18, 20, 21, 29–31], with an established range or cut-off point of 8–10 mm^2^ [32–42]. However, only a few authors have included longitudinal evaluations [1, 34, 40, 43], but never in healthy or control individuals; furthermore these studies may generate controversy due to the poor definition of the images that do not allow a good discrimination of the rims.

We studied CSA reference values for the ulnar nerve (UN) as well as EDX, and most importantly longitudinal US evaluation, in a group of healthy subjects.

## Methods

### Participants

We studied 76 arms from 38 asymptomatic volunteers with normal EDX parameters. Written informed consent was obtained from all subjects. The study was approved by the institutional ethics committee. Two age groups were formed as a median split: over and under 40 years old. Individuals with symptoms and signs of UNE, a medical history related to neuropathy or any disease of the peripheral nervous system, as well as those with abnormal findings on EDX were excluded from the study.

### EDX

Nerve conduction studies (NCS) were performed in all subjects by an expert neurophysiologist following the recommendations of the American Association of Electrodiagnostic Medicine and the International Federation of Clinical Neurophysiology [1, 20]. A Nihon-Kodden electromyograph (NihonKohden, Tokyo, Japan, 2010) was used for measurements. The examiners performed radial, median and ulnar motor NCS, the latter with stimulation at three points, wrist, below elbow (volar crease) and above elbow. The ME was used as a reference: 3 cm distal and 7 cm proximal to the ME (10 cm between the 2 stimulation points). The recording electrodes were placed at the *abductor digiti minimi* and *first dorsal interosseous* muscles. Orthodromic distal sensory nerve action potential (SNAP) were performed and measured at median and ulnar nerves. Motor conduction velocity (MCV), sensory conduction velocity (SCV), and amplitude of the compound motor action potential were assessed.

### Ultrasonography

**Fig. 1:**
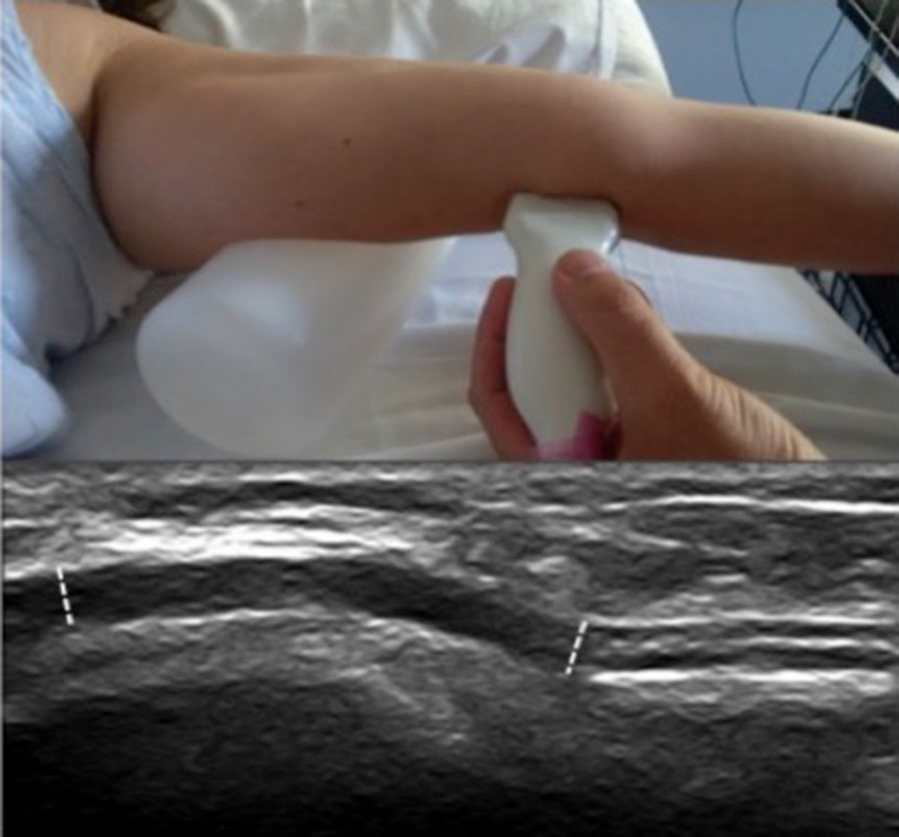
Top: position of the arm for the longitudinal ultrasound examination. Bottom: normal ultrasound of the UN at the elbow in the longitudinal evaluation; white lines indicate ASD, distal (right) and proximal (left)

**Fig. 2:**
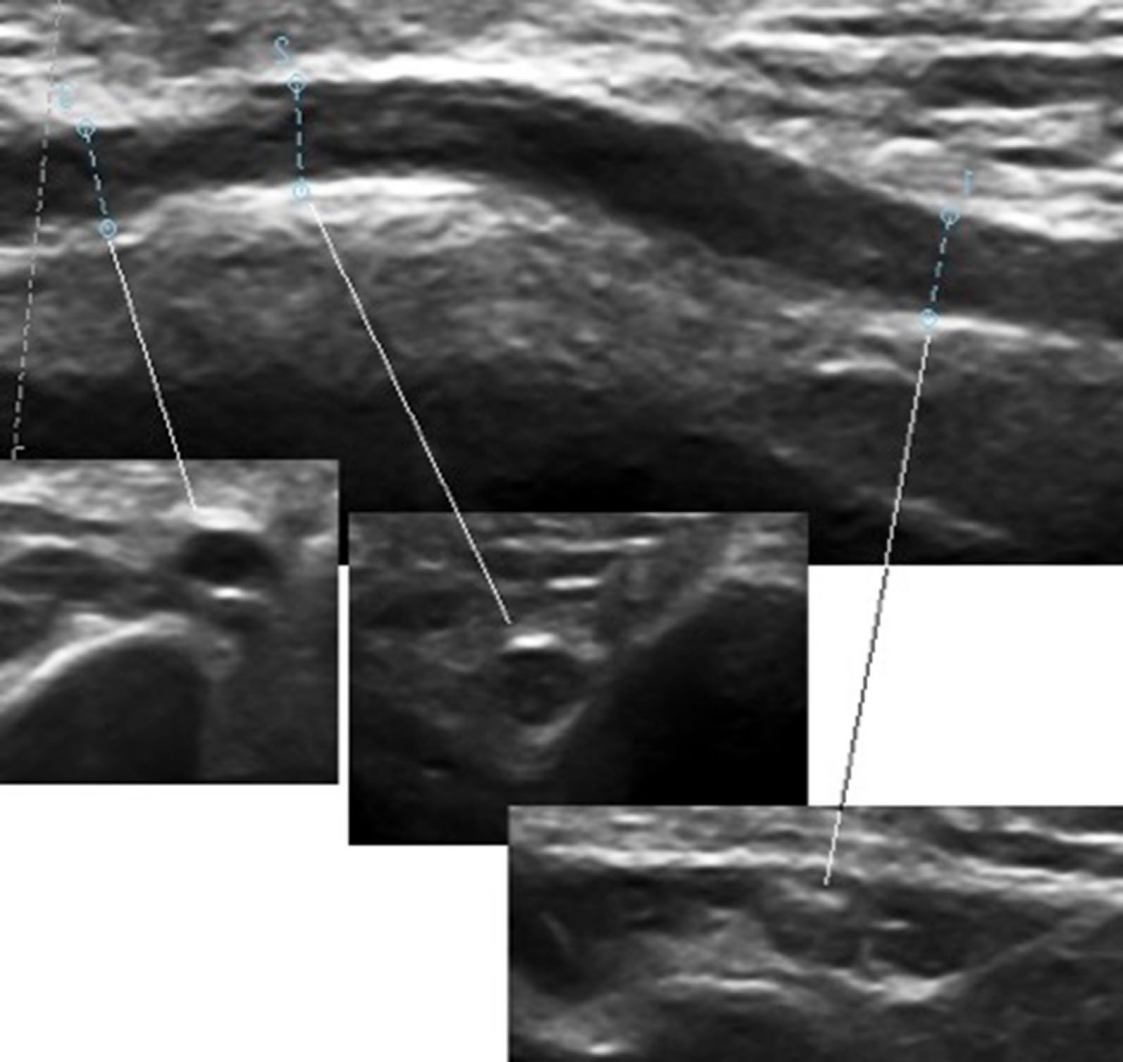
Above, horizontal elbow position. Ultrasonographic evalution of ulnar groove. Left proximal segment, right distal segment Below, transversal evaluations. Right, distal segment (ulnar tunnel); middle and left proximal segment (ulnar groove)

The US evaluation was performed by an expert sonographer. A linear probe with frequencies of 10–18 MHz (*Telemed Inc., Lithuania, 2017*) was used. The cross-sectional evaluation of distal and proximal CSAs was obtained at the UG. Longitudinal evaluation of the antero-superior diameters (ASD) (Fig. 1), or what is the same the axis, was performed by placing the central point of the probe at the ME, allowing assessment of the nerve course from 2 cm distal to 2 cm proximal to the ME, and identifying the different anatomical landmarks such as the medial humerus, the *triceps brachii* and the exit of the UG and ulnar tunnel. Subjects were studied in supine position and the examined limb was placed in extension and passive supination with a cylindrical structure under the mid-proximal third of the arm (Fig. 2), corrected by another distal support if there was hyperextension of the elbow. Both the CSA and the ASD were measured within the thickness of the epineurium. We analyzed CSA and ASD in the UG at the 2 locations mentioned above to the ME, distal (d) or minor axis, and proximal (p) or maior axis: dCSA, pCSA, dASD and pASD, respectively, the difference between both points, the neural echostructure and the morphology of the epineurium.

### Statistical analysis

Statistical analysis was performed using SPSS 11.5 (IBM, SPSS Inc, 2003, Chicago, IL, USA). The distribution of the variables studied was analyzed by the Kolmogorov–Smirnov test. Normally distributed variables were compared by the Student’s t-test whereas non-normally distributed variables were compared by the Mann-Whitney U test. Continuous variables were expressed as a mean ± standard deviation. Correlations between variables were analyzed using Pearson’s correlation coefficient, followed by chi-square or Fisher’s exact tests, as appropriate. If applicable, a *p* value < 0,05 was considered statistically significant.

## Results

The values ​​obtained from 76 upper limbs (38 right and 38 left limbs) and 38 control subjects (28 women and 10 men) were analyzed. The mean age (mean ± SD) was 46 ± 14 years (range 22–81). The 90% of the subjects were right-handed and all of them were caucasian.

**Fig. 3:**
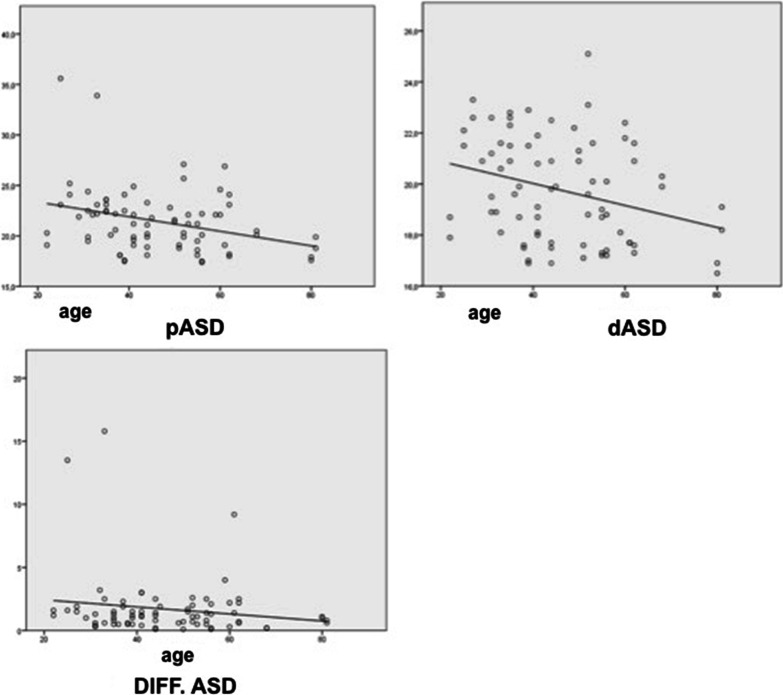
Correlation between antero-superior elbow diameter (longitudinal evaluation) and age. Top left: proximal location. Top right: distal location. Bottom: difference between diameters

All US variables were analyzed (Fig. 3, Table 1). The values ​​of both variables in the proximal region of the UG were significantly higher than those in the distal region (*p* < 0.04). When we compared the values ​​by age group, the morphological values ​​studied by the ASD were higher in the younger age group, especially the pASD (*p* < 0.02) (Fig. 2); pCSA did not show any significant difference between both groups (Table 1). No significant differences were found by sex, hand dominance, weight, height or any other demographic variable.

**Table 1:** US parameters and age groups

US parameters	Total subjets	Age groups, means ± SD (CSA mm^2^, ASD mm)		
		18–40 year	40 year and above	*P* value
dASD	1.97 ± 0.20	2.0 ± 0.1	1.9 ± 0.2	0.04
pASD	2.14 ± 0.32	2.2 ± 0.4	2.0 ± 0.2	0.02
Dif ASD	0.10 ± 0.02	0.21 ± 0.35	0.14 ± 0.14	0.1
dCSA	7.00 ± 1.03	6.7 ± 0.9	7.1 ± 1.0	0.05
pCSA	7.85 ± 1.46	7.5 ± 1.5	8.0 ± 1.4	0.1

*ASD* anterosuperior diameter, *d* distal, *p* proximal, *ASD diff* anterosuperior diameter difference, *CSA* cross-sectional area, *mm*, millimeters

The EDX variables were obtained from all subjects. Similarly to the US variables, we found several parameters significantly varying with age. The older group showed lower values for SNAP, amplitude, SCV and MCV, ​​with high statistical significance (*p* < 0.003), but not for ASD (*p* < 0.6). No correlations were found for the other demographic variables.

## Discussion

In this study we used US to assess the morphological parameters of the UN at the elbow in a control population. Special attention was paid to the longitudinal view in order to determine normative values, especially for the ASD along the entire trajectory of the UG but CSA measurement is probably more reliable for quantitative evaluation. We also evaluated other US and EDX parameters by correlating them to demographic variables (sex, age, dominant side).

Regarding the US longitudinal view, we found a larger UN size in the most proximal region of the UG, as we compare the pASD (2.1 mm ± 0,3) with the dASD (1.9 mm ± 0.2). This observation is consistent with previously reported US findings, although they were based on cross-sectional evaluations [2, 3, 8, 10, 12, 13, 18, 21, 29–31]. This suggests a slight anatomical narrowing in the most distal part of the UG, which is the path where the nerve crosses between the two attachments of the *flexor carpi ulnaris* muscle and the aponeurosis that they share in the upper part, the Osborne’s ligament. On the other hand, the most proximal segment of the nerve at the retroepicondylar groove is less narrow and is surrounded by a more distensible tissue, which allows greater morphological deformation and an US view more useful for diagnosis. Often nerve is thickened there also in asymptomatic subjects [44, 45], and this is other reason for interest in our study, to define an anatomical cut-off point to assess pathological deformity.

We have hardly found any studies that evaluate the UN in longitudinal plane [31, 34, 40, 43]. Yang et al. [31] studied 65 patients with UNE using US in a longitudinal view before surgery, later confirming the results by direct observation. As in our series, they found larger dimensions for the UN at the proximal region of the UG, next to the ME, when compared to the distal region. This does not seem to be relevant from the anatomical point of view, because in UNE a proximal thickening of this segment of the UN constitutes a habitual characteristic sign, as is also described in the study by Beekman et al. [40]. In our study, UN size was only related to age. We found a slightly smaller UN size in individuals older than 40 years, contrary to that reported by some authors [23, 25, 26, 28]. In those studies, larger size was particularly patent in the elderly, perhaps due to a greater probability of presenting asymptomatic entrapment or accumulation of trauma caused by repetitive external compression. In our study, the ≥ 40-year-old group included few elderly subjects, which could partly explain the discrepancy between findings. However, there is no apparent reason why these nerves outside the entrapment sites should be larger in the elderly. The smaller UN size that we found in the older individuals is likely related to degenerative physiological processes such as neuronal and axonal loss and the corresponding reduction in neural volume.

The size of the nerves can also vary in relation to weight and height, which could bias the establishment of normative values​. This possible pitfall can be easily avoided by using ratios or differences between values ​​in the same subject at different points along the nerve path. We did not find the CSA-gender correlation reported by Won et al. [19]. However, in agreement with our results, other authors also failed to find such correlation. The US assessment of the nerve structure provides great sensitivity when examining entrapments, especially morphological alterations of intraneural structure and dimensions of epineurium. The US has recently become a useful technique thanks to recent advancements that allow levels of discrimination even greater than 1 mm in surface images.

Therefore, US can be considered a rapid and highly sensitive method for detecting nerve entrapments. Under normal circumstances, the UN presents certain peculiarities with respect to some of the intraneural morphology parameters most commonly assessed, such as the fascicular pattern in the cross-sectional plane, or the fibrillar pattern in the longitudinal plane. Thus, in the UN at the elbow, only a few fascicles are visible in cross-section. This nerve is also highly hypoechoic and slightly larger than 1 mm^2^ at the retroepicondylar region compared to distal view of the nerve through the tunnel [36, 37, 39–42] which allows a greater elasticity and deformability in a segment where joint mobility is very important. The epineurium is more hyperechoic and slightly thickened, especially in its postero-inferior portion. All these signs can be seen in a single plane from the distal to proximal end in a longitudinal plane with the arm in passive extension. This allows a more complete evaluation of possible deformation, narrowing or thickening of the nerve, as well as its intraneural content across this segment. The influence of elbow position on US measurements has been a matter for discussion. Thoirs et al. [12] demonstrated that the elbow position of the patient affected the US measurement of the UN diameter. We carried out our study with the arm in abduction (60°) and the elbow in passive extension (180º), since this arrangement causes minimal nerve deformation. Forced flexion (≥ 90°) due to certain anatomical factors, such as the relative fixation of the nerve to muscular and ligamentous structures at the distal region (both at the entry point and, especially, at the exit point), produces superficialization, elongation and possible compression of the nerve. In this regard, Padua et al. [46] demonstrated that prolonged flexion of the elbow induced nerve dysfunction as assessed by EDX, specifically a decrease in MCV; this is probably due to compressive or ischemic effects on the nerve; similar findings were obtained by Lee et al. [47]. In fact, the association between UNE and fixed flexion positions as an etiological factor is well known [23, 48]. Some US studies have even shown that flexion of the elbow deforms the nerve (narrowing it up to 11% of the CSA) and increases the pressure in the distal third of the UG up to 200 mm Hg [49, 50]. This deformity is associated with backward displacement of the olecranon and increased secondary pressure from the humero-ulnar aponeurotic arch. In our study, placing the limb in extension and passive supination allowed for a relaxed and superficial exposure resulting in absence of nerve deformation and dysfunction, as well as good resolution when evaluating the nerve in the UG.

It is uncertain that the UN might also be lax on full elbow extension, making longitudinal view more difficult and less reliable, on the contrary, this position is more anatomical and allows a more normal disposition of the nerve than when the joint is flexed because the nerve is tractioned and relatively deformed even causing nerve dysfunction [47–52]. In addition, in the extension position, the length of the nerve is better estimated, which eliminates artefactual assessments of the VCM at the elbow. The EDX was performed in the same position to standardize the results and for the above mentioned physiological reasons.

Most of the studies dealing with the characterization of the UN (as well as many other nerves) with US at the elbow, employ cross-sectional planes, particularly when measuring CSA. In recent years, several studies have been published aimed at determining the CSA cut-off point for the diagnosis of UNE [2, 3, 8, 10–13, 18, 21, 29–31, 35, 37–42]. The most consensual value is 9 mm^2^, with a sensitivity and specificity somewhat higher than 88%, although it has also been suggested that it may be closer to 8 mm^2^. Another way to determine the CSA, as proposed by Bayrak et al. [50], is to compare the distal and proximal ME areas in the UG, which results in a ratio greater than 1.5. In fact, these same authors found 100% sensitivity with a cut-off point for the area set at 8.3 mm^2^ for the ME. In addition, they proposed that the level of nerve injury could be determined by evaluating the CSA using a 12 MHz transducer probe, establishing a limit of 11.2 mm ^2^ for mild injuries, 15.8 mm^2^ for moderate and 18.3 mm^2^ for severe neuropathies, with a sensitivity of 88%. This method has been open to criticism for being so precise and so narrow in scope. We found a great variability in the morphology of the pathological UN. This is probably due to the absence of tissues that limit the thickening that surrounds the nerve throughout most of the retroepicondylar segment; in fact, the longitudinal evaluation is particularly illustrative of this phenomenon.

## Conclusion

We demonstrate that longitudinal US evaluation of the UN at the elbow offers good morphological resolution, is easy and rapid to perform, as well as a wide and complete view of the UN at the principal UG segment and the usual and varied signs in a single evaluation. We believe that the longitudinal US evaluation of the UN at the elbow should be incorporated, but follow-up studies in UNE are needed to determine the real diagnostic value of US and its different manifestations.

## Data Availability

Not applicable.

## Abbreviations

UNE: Ulnar neuropathy at elbow
EDX: Electrodiagnostic tests
US: Ultrasonography
CSA: Cross sectional area
UG: Ulnar groove
ME: Medial epycondile
UN: Ulnar nerve
NCS: Nerve conduction studies
SNAP: Sensory nerve action potential
SCV: Sensory conduction velocity
MCV: Motor conduction velocity
ASD: Antero-superior diameters
d: Distal
p: Proximal
